# Rapid detection of multiple resistance genes to last-resort antibiotics in *Enterobacteriaceae* pathogens by recombinase polymerase amplification combined with lateral flow dipstick

**DOI:** 10.3389/fmicb.2022.1062577

**Published:** 2023-01-05

**Authors:** Chenze Lu, Jingwen Wang, Leiming Pan, Xiuying Gu, Wenjing Lu, Di Chen, Cen Zhang, Qin Ye, Chaogeng Xiao, Pengpeng Liu, Yulong Tang, Biao Tang, Guangrong Huang, Jiehong Fang, Han Jiang

**Affiliations:** ^1^Key Laboratory of Specialty Agri-Products Quality and Hazard Controlling Technology of Zhejiang Province, College of Life Sciences, China Jiliang University, Hangzhou, Zhejiang, China; ^2^Zhejiang Hongzheng Testing Co., Ltd, Ningbo, Zhejiang, China; ^3^Zhejiang Gongzheng Testing Center Co., Ltd, Hangzhou, Zhejiang, China; ^4^Institute of Food Science, Zhejiang Academy of Agricultural Sciences, Hangzhou, Zhejiang, China; ^5^Key Laboratory of Biosafety Detection for Zhejiang Market Regulation, Zhejiang Fangyuan Testing Group LO.T, Hangzhou, Zhejiang, China; ^6^Hangzhou Tiannie Technology Co., Ltd, Hangzhou, Zhejiang, China; ^7^State Key Laboratory for Managing Biotic and Chemical Threats to the Quality and Safety of Agro-Products and Institute of Agro-Product Safety and Nutrition, Zhejiang Academy of Agricultural Sciences, Hangzhou, Zhejiang, China

**Keywords:** recombinase polymerase amplification, lateral flow dipstick, *Enterobacteriaceae*, rapid detection, resistance genes to last-resort antibiotics

## Abstract

The worrying emergence of multiple resistance genes to last-resort antibiotics in food animals and human populations throughout the food chain and relevant environments has been increasingly reported worldwide. *Enterobacteriaceae* pathogens are considered the most common reservoirs of such antibiotic resistance genes (ARGs). Thus, a rapid, efficient and accurate detection method to simultaneously screen and monitor such ARGs in *Enterobacteriaceae* pathogens has become an urgent need. Our study developed a recombinase polymerase amplification (RPA) assay combined with a lateral flow dipstick (LFD) for simultaneously detecting predominant resistance genes to last-resort antibiotics of *Enterobacteriaceae* pathogens, including *mcr-1*, *bla_NDM-1_* and *tet(X4)*. It is allowed to complete the entire process, including crude DNA extraction, amplification as well as reading, within 40 min at 37°C, and the detection limit is 10^1^ copies/μl for *mcr-1*, *bla_NDM-1_* and *tet(X4)*. Sensitivity analysis showed obvious association of color signals with the template concentrations of *mcr-1*, *bla_NDM-1_* and *tet(X4)* genes in *Enterobacteriaceae* pathogens using a test strip reader (*R*^2^ = 0.9881, *R*^2^ = 0.9745, and *R*^2^ = 0.9807, respectively), allowing for quantitative detection using multiplex RPA-LFD assays. Therefore, the RPA-LFD assay can suitably help to detect multiple resistance genes to last-resort antibiotics in foodborne pathogens and has potential applications in the field.

## 1. Introduction

Imprudently using antibiotics in medicine and farming has resulted in increasingly severe bacterial resistance problems around the world (Fu et al., 2022). According to the World Health Organization (WHO), antibiotic resistance remarkably threatens food security, and global health and development (World Health Organization, 2022). Multidrug resistance (MDR) occurs when bacteria obtain resistance to three or more categories of antibiotics, causing them to pose more urgent threats (Cheng et al., 2019). Among the currently available antibiotics, carbapenems, colistin as well as tigecycline are last-resort antibiotics for managing MDR bacterial infections, especially those caused by *Enterobacteriaceae* (Tang et al., 2021). Unfortunately, the worrying emergence of the predominantly plasmid-borne colistin resistance gene *mcr-1*, carbapenem resistance gene *bla_NDM-1_* as well as tigecycline resistance gene *tet(X4)* in *Enterobacteriaceae* pathogens from different sources, such as animals, food and humans, has increasingly been reported in different continents (Singh et al., 2018; Zhong et al., 2019; Anyanwu et al., 2022; Lu et al., 2022; Zhang et al., 2022). Additionally, *tet(X)* genes have regularly been reported to coexist with *bla_NDM-1_* and/or *mcr-1* genes (Chen et al., 2019; Sun C. et al., 2019), and such plasmid-borne antibiotic resistance genes (ARGs) are capable of transferring between epidemic strains of *Enterobacteriaceae* (Leshaba et al., 2022). Thus, a fast, efficient and accurate detection method for simultaneously screening and monitoring the above-mentioned ARGs in *Enterobacteriaceae* pathogens has become urgently needed for managing the dissemination of resistance in food animal and human populations throughout the food chain and relevant environments.

The most widely used molecular-based method for MDR gene detection is polymerase chain reaction (PCR), including conventional PCR (Adekunle et al., 2021; Cooper et al., 2021), real-time quantitative PCR (Park et al., 2021; Tolosi et al., 2021) and digital PCR (Xu et al., 2021; Merino et al., 2022). However, most farms in China are equipped with laboratories capable of isolating bacteria from environmental samples, but are generally not equipped with complex large-scale instruments such as qPCR (Ma et al., 2020). A method which is simpler to operate and less dependent on instruments is urgently needed. To date, researchers have developed novel simple, rapid and efficient and cost-effective molecular biology techniques, such as isothermal nucleic acid amplification, as alternative protocols for the rapid on-site evaluation of MDR genes (Wong et al., 2018; Li et al., 2019; Tran et al., 2022). Recombinase polymerase amplification (RPA) acts as a common nucleic acid isothermal amplification technologies first developed by Piepenburg et al. (2006). It adopts a phage recombinase for forming complexes with oligonucleotide primers, thereby facilitating oligonucleotide primers to bind to homologous sequences of the double-stranded DNA molecules (Lobato and O'Sullivan, 2018). It is allowed to accomplish amplification within 30 min at a constant low temperature around 37°C by combining with a single-stranded DNA binding protein and a strand-displacing polymerase (Lobato and O'Sullivan, 2018). In addition, only a few copies of target DNA are initially needed, and highly specific DNA amplification at detectable levels can be achieved within a short time (Kersting et al., 2014). Furthermore, multiplexing RPA amplifications in the same solution is feasible, despite its strong dependence on the amplicon size, target sequences, as well as primer design (Kersting et al., 2014). Detection of RPA products is mainly completed *via* probe-based florescence (Kim and Lee, 2016), gel electrophoresis (Cao et al., 2018), or visualization by nucleic acid lateral flow dipstick (LFD) immunoassays (Xu et al., 2018). Among these, LFD immunoassays are simple, fast (~5 min), cost-effective and accurate for detection of amplified products and are more suitable for point-of-care testing (Lin et al., 2022). Additionally, multiplex LFD immunoassays that analyze multiple targets simultaneously have emerged in several research fields. The most common solution is to prepare several test lines on one test strip (Di Nardo et al., 2021). For instance, Cavalera et al. reported a multi-target LFD immunoassay with two test lines that bound to several classes of immunoglobulins, which realized detection of total antibodies against SARS-CoV-2 (Cavalera et al., 2021). Such designs further reduce cost and increase throughput of detection, which is vital to the study of MDR genes. Recently, to meet increased accuracy requirements for quantitative detection, test strip readers (Li et al., 2019) and image software (Wang et al., 2021) have been employed to scan and evaluate the color signals of LFD.

The study focused on developing an RPA-LFD method for simultaneously identifying the highly prevalent genes *mcr-1*, *bla_NDM-1_* and *tet(X4)* in certain *Enterobacteriaceae* pathogens isolated from different food and animal fecal samples. In addition, a TSR-200 test strip reader (Allsheng Instruments Co., Ltd., Hangzhou, China) assisted in evaluating color signals to use LFD for accurate quantitative analysis. This allows for immediate monitoring of MDR genes in pathogenic bacteria in the field and will inform risk assessment and remediation strategies in a timely manner.

## 2. Materials and methods

### 2.1. Bacterial strains

We used 19 MDR *Enterobacteriaceae* strains, including 14 *Escherichia coli* strains, 1 *Escherichia fergusonii* strain, 2 *Klebsiella pneumoniae* strains and 2 *Salmonella* spp. strains, for confirming the multiplex RPA-LFD assay specificity and its application in the field in our study. Detailed information on isolates is shown in Supplementary Table S1. The three recombinant *E. coli* strains containing standard plasmids carrying *mcr-1*, *bla_NDM-1_* or *tet(X4)* genes were previously constructed in our laboratory to be used as reference strains for optimizing the reaction system and analyzing the sensitivity (Supplementary Table S1).

### 2.2. DNA extraction

The standard DNA plasmids that carried *mcr-1*, *bla_NDM-1_* and *tet(X4)* genes were extracted from recombinant *E. coli* Top10-pUC-*mcr-1, E. coli* Top10-pUC-*bla_NDM-1_* and *E. coli* Top10-pUC-*tet(X4)* using a QIAGEN Plasmid Mini Kit (QIAGEN, Hilden, Germany) according to the manufacturer’s instructions.

The DNA of all *Enterobacteriaceae* strains was prepared using the previously described boil-up protocol with some modifications (Bordin et al., 2019). Briefly, a 1 μl solution of each *Enterobacteriaceae* strain was added into 30 μl 10 mM Tris buffer, boiled for 5 min in a metal bath, cooled on ice for 2 min together with 2 min of centrifugation at 12, 000 × *g*. RPA-LFD assays took the supernatant as the DNA template.

### 2.3. Design of RPA primers and LF probes

The ARG sequences of *mcr-1* (KP347127.1), *bla_NDM-1_* (NG_049326.1) and *tet(X4)* (MK134376.1) came from GenBank.^1^ Multiple gene sequence alignments of *mcr-1*, *bla_NDM-1_* and *tet(X4)* available from GenBank were analyzed using ClustalW. Based on the conserved sequences, Primer Premier 5 served for designing RPA primers and LF probes following the instruction manual provided by TwistDX (Cambridge, United Kingdom). The specificity exhibited by all RPA primers and LF probes was evaluated *in silico* using the BLASTn tool of the NCBI database. In this study, the 5′ ends of the reverse primers for *mcr-1*, *bla_NDM-1_* and *tet(X4)* were all labeled with digoxin. The 5′ ends of the LF probes for *mcr-1*, *bla_NDM-1_* and *tet(X4)* were labeled with biotin, cyanine 5 (Cy5) and carboxytetramethylrhodamine (TAMRA), respectively. The 3′ end of the LF probe was modified with 3′ spacer C3. The inside of the LF probe was modified with an internal abasic nucleotide analog dSpacer (tetrahydrofuran, THF) (Table 1). GENEray Biotechnology (Shanghai, China) helped to synthesize all PRA primers and LF probes.

**Table 1:** Information of RPA primer and LF probe.

Name	Sequences (5′-3′)	Target gene
*mcr-1*-F1	CCCTACAGACCGACCAAGCCGAGACCAAGG	*mcr-1*
*mcr-1*-R1-Dig	Digoxin-CTGGCATAATGACTGCTGAACGCCACCACAG	
*mcr-1*-P-Bio	Bio-GGGTGTGCTACCAAGTTTGCTTGTGGCTTT-dSpacer(THF)-GTTAAGGTGGATTATCCG-C3-Spacer	
*bla_NDM-1_*-F1	CAGTCGCTTCCAACGGTTTGATCGTCAGGG	*bla_NDM-1_*
*bla_NDM-1_*-R1-Dig	Digoxin-CCAGCCATTGGCGGCGAAAGTCAGGCTGTG	
*bla_NDM-1_*-P-Cy5	Cy5-CCAGACCGCCCAGATCCTCAACTGGATCAAGC-dSpacer(THF)-GGAGATCAACCTGCCGGTC-3’C3 Spacer	
*tet(X4)*-F1	TACCCATAACGATGATTGGAGATGCTGCTC	*tet(X4)*
*tet(X4)*-R1-Dig	Digoxin-GCTTCTTTGCCATAGATAAACATTTGCTGT	
*tet(X4)*-P-TAMRA	TAMRA-GCGTAAACAGCGGGTTGATGGATGCCTTGA-dSpacer(THF)-ATTGTCGGATAATCTGACCAA-3’C3 Spacer	

### 2.4. LFD strip preparation and RPA-LFD assay visualization

LFD strips were prepared *via* methods described in previous studies with some modifications (Ma et al., 2020; Jin et al., 2022). Briefly, the LFD strips were composed of continuous superposition: sample pad, conjugate pad with gold nanoparticles (AuNPs), nitrocellulose filter (NC) membrane, adsorption pad as well as backing card. The AuNPs were synthesized using sodium citrate tannin reduction, labeled by anti-digoxin monoclonal antibody (mAb) and sprayed onto conjugate pads. We prepared the three test lines (T-lines) using anti-biotin mAb (0.65 mg/ml, for detection of *mcr-1* in T1), anti-Cy5 mAb (0.3 mg/ml, for detection of *bla_NDM-1_* in T2) and anti-TAMRA mAb (0.3 mg/ml, for detection of *tet(X4)* in T3). Immobilization of an anti-mouse polyclonal secondary antibody (pAb, 2.0 mg/ml) on the control line (C-line) was completed. The immobilized NC membrane received 12 h of drying treatment at 37°C and a cutter was used to cut it into 2.5 mm wide strips. We stored the assembled LFD test strips in a vacuum bag at room temperature before use. We diluted the RPA amplification products 50 times (mixing 2 μl product with 98 μl running buffer containing phosphate buffered saline and 3% Tween20) and pipetted them onto the LFD strips. The labeled RPA products were then migrated by capillary action. A test strip reader assisted in scanning as well as calculating the T1–T3 lines and C-line intensities within 5 min.

### 2.5. Single RPA-LFD assays

Single RPA assays were performed to test the validity of the primer/probe sets. The reaction components and conditions were set following the operating manual regarding the TwistAmp nfo kits (TwistDX) with some modifications. Each reaction had 29.5 μl rehydration buffer to dissolve the freeze-dried enzyme pellet, 2.1 μl of each reverse and forward primer (10 μM), 0.6 μl LF probes (10 μM), 2.5 μl magnesium-acetate (280 mM), 2 μl DNA template and 11.2 μl nuclease-free water. The tube was mixed by inversion and brief centrifugation, and the single RPA amplification reaction proceeded for 20 min at 37°C. The RPA amplification products were diluted 50 times and pipetted onto LFD strips. The T1–T3 lines and C-line intensities were analyzed within 5 min.

### 2.6. Optimization of multiplex RPA-LFD assays

Based on the single RPA assay, concentrations of the primer/probe sets, reaction temperature, incubation time as well as magnesium-acetate concentration were optimized for multiplex RPA assays to simultaneously amplify *mcr-1*, *bla_NDM-1_* and *tet(X4)* genes. First, the primer/probe concentrations were evaluated in the following ratios: (1) high primer/probe concentrations (*mcr-1* 0.42 μM/0.12 μM, *bla_NDM-1_* 0.42 μM/0.12 μM and *tet(X4)* 0.42 μM/0.12 μM); (2) medium primer/probe concentrations (*mcr-1* 0.30 μM/0.09 μM, *bla_NDM-1_* 0.30 μM/0.09 μM and *tet(X4)* 0.30 μM/0.09 μM; *mcr-1* 0.42 μM/0.12 μM, *bla_NDM-1_* 0.30 μM/0.09 μM and *tet(X4)* 0.15 μM/0.06 μM; *mcr-1* 0.30 μM/0.09 μM, *bla_NDM-1_* 0.42 μM/0.12 μM and *tet(X4)* 0.15 μM/0.06 μM; *mcr-1* 0.15 μM/0.06 μM, *bla_NDM-1_* 0.30 μM/0.09 μM and *tet(X4)* 0.42 μM/0.12 μM; *mcr-1* 0.30 μM/0.09 μM, *bla_NDM-1_* 0.42 μM/0.12 μM and *tet(X4)* 0.30 μM/0.09 μM); and (3) low primer/probe concentrations (*mcr-1* 0.15 μM/0.06 μM, *bla_NDM-1_* 0.15 μM/0.06 μM and *tet(X4)* 0.15 μM/0.06 μM).

Second, multiplex RPA reactions were performed at a 30–50°C temperature range (30, 35, 37, 39, 45 and 50°C). Finally, we assessed the optimal incubation time from 2.5 to 30 min.

### 2.7. Specificity and sensitivity analyses of multiplex RPA-LFD assays

For specificity analysis regarding the multiplex RPA-LFD assay, DNA from the recombinant *E. coli* strains containing *mcr-1*, *bla_NDM-1_* or *tet(X4)* and *E. coli* ATCC25922 (quality control reference strain in all antimicrobial susceptibility tests) was extracted and added to the optimized reaction system.

For sensitivity analysis, the three standard recombinant plasmids for the *mcr-1*, *bla_NDM-1_* and *tet(X4)* genes were chosen as the standard DNA templates. Concentrations of the standard DNA were determined using a BioDrop μLITE (BioDrop, Cambridge, United Kingdom). Below is the calculation formula of the copy number:


$$\mathrm{Copy number}\;\left(\mathrm{copies}/\mu L\right)=\left(X/\left[a\times 660\right]\;\right)\times 6.02\times 10^{23}$$

where X is the concentration of the standard DNA (g/μl) measured at 260 nm wavelength and a is the number of base pairs of the standard DNA molecule (bp).

The diluted standard DNA was prepared by serial 10-fold dilution in the range of 10^0^ copies/μl to 10^7^ copies/μl. Serially diluted plasmids containing the *mcr-1*, *bla_NDM-1_* and *tet(X4)* genes served as the DNA templates, respectively, for sensitivity analysis of the multiplex RPA-LFD assay.

Quantitative analysis of the *mcr-1*, *bla_NDM-1_* and *tet(X4)* genes in *Enterobacteriaceae* was also performed. The TSR-200 test strip reader (Allsheng Instruments Co. Ltd.) assisted in reading the RPA-LFD assay test results. Standard curves were established for the *mcr-1*, *bla_NDM-1_* and *tet(X4)* genes in *Enterobacteriaceae* strains considering the T/C value of the LFD strips and the logarithm of the copy number of the corresponding target resistance gene.

### 2.8. Application analysis of multiplex RPA-LFD assays

*Enterobacteriaceae* strains for the application of multiplex RPA-LFD assays are listed in Supplementary Table S1. All isolates were confirmed to carry *mcr-1*, *bla_NDM-1_* and/or *tet(X4)* genes by sequencing. The triplex RPA-LFD assays were compared with conventional PCR methods as described previously (Xu et al., 2018; Ji et al., 2020; Ayfan et al., 2021).

### 2.9. Data analysis

We scanned the intensities exhibited by the T-line and C-line, and calculated the T/C value using a TSR-200 test strip reader (Allsheng Instruments Co. Ltd.). We carried out all experiments in triplicate, and the results are in the form of mean ± standard deviation. SPSS 20.0 (IBM Corp., Armonk, NY, United States) together with Origin 2021 (OriginLab, Northampton, MA, United States) served for the statistical analysis.

## 3. Results

### 3.1. RPA-LFD assay strategy

**Figure 1:**
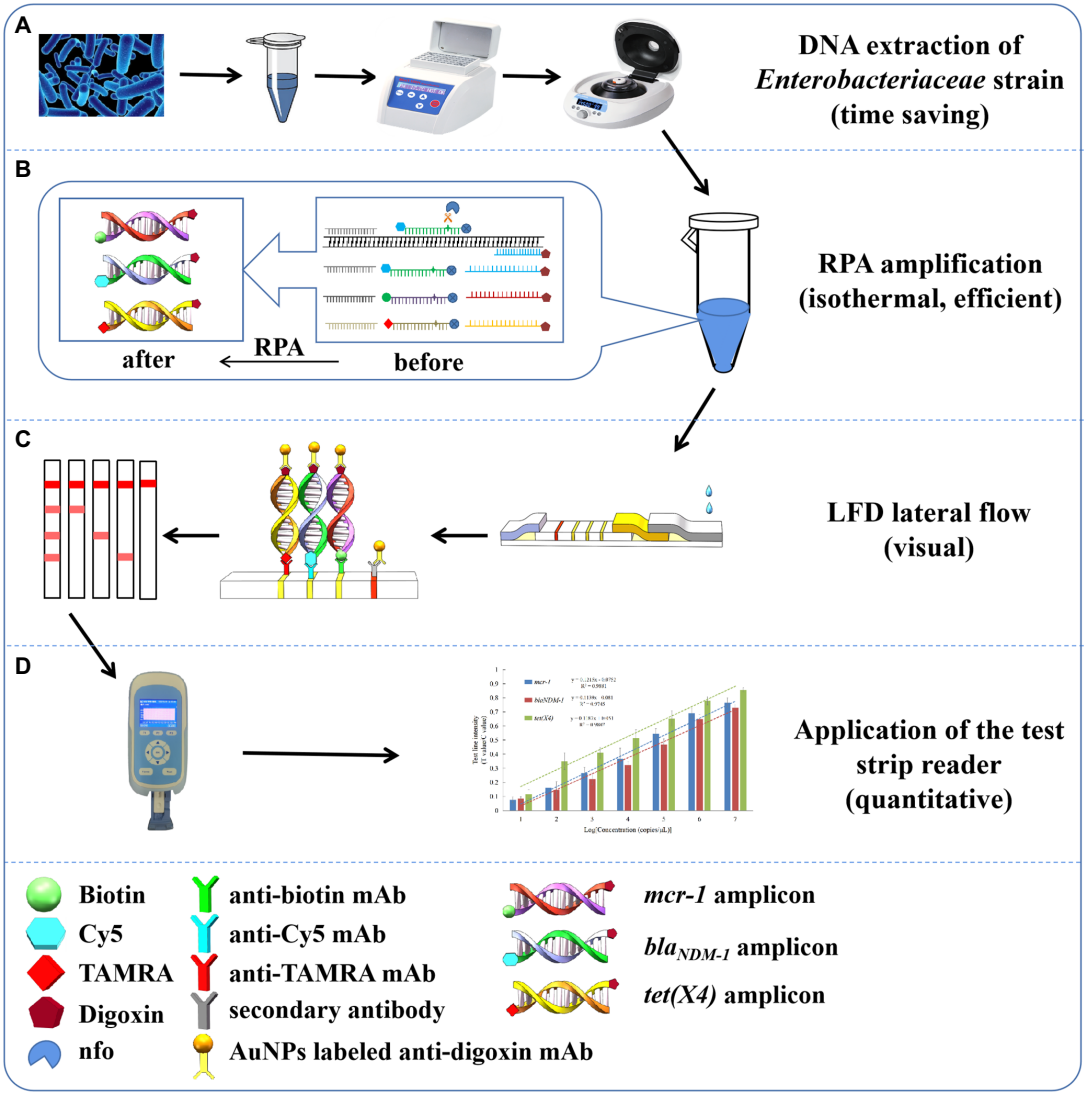
RPA-LFD assay strategy. **(A)** DNA extraction of *Enterobacteriaceae* strains; **(B)** RPA amplification; **(C)** LFD lateral flow; **(D)** quantitative analysis.

Here, a fast boil-up method for DNA extraction from *Enterobacteriaceae* strains was applied accrding to previous studies with some modification (Bordin et al., 2019; Liu et al., 2022). Compared with the commercial DNA extraction kits (taking around 30 min or more, Dimitrakopoulou et al., 2020), our method significantly reduces extraction time (taking around 10 min for the entire extraction process; Figure 1A).

A TwistAmp nfo kit served for conducting the triplex RPA amplification to generate three target amplicons. Various chemical groups were labeled for designing primers and LF probes compatible with the TwistAmp nfo technology. The resulting double-stranded DNA amplicons for the *mcr-1* target were digoxin- and biotin-labeled, the amplicons for the *bla_NDM-1_* target were digoxin- and Cy5-labeled and the amplicons for the *tet(X4)* target were digoxin- and TAMRA-labeled. These labeled double-stranded DNA amplicons could be detected by the LFD assay (Figure 1B).

On the LFD test strip, when amplicons moved on the conjugated pad, the digoxin-labeled amplicons combined with AuNP-labeled anti-digoxin mAb. With further migration, the biotin-, Cy5- and TAMRA-labeled amplicons combined with the AuNP-labeled anti-digoxin mAb were captured by a corresponding mAb coated on the T-lines. The uncaptured AuNP-labeled anti-digoxin mAb continued moving and was captured by the pAb coated on the C-line (Figure 1C). Using the test strip reader, quantitative analysis was then achieved. First, the test strip reader served for scanning the intensities exhibited by T-line and C-line, and T and C values were calculated. The T/C value was positively correlated with the concentration of target DNA. Standard curves were plotted considering the T/C value and the copy number logarithm of target DNA (Figure 1D).

### 3.2. Optimization of triplex RPA amplification conditions

**Figure 2:**
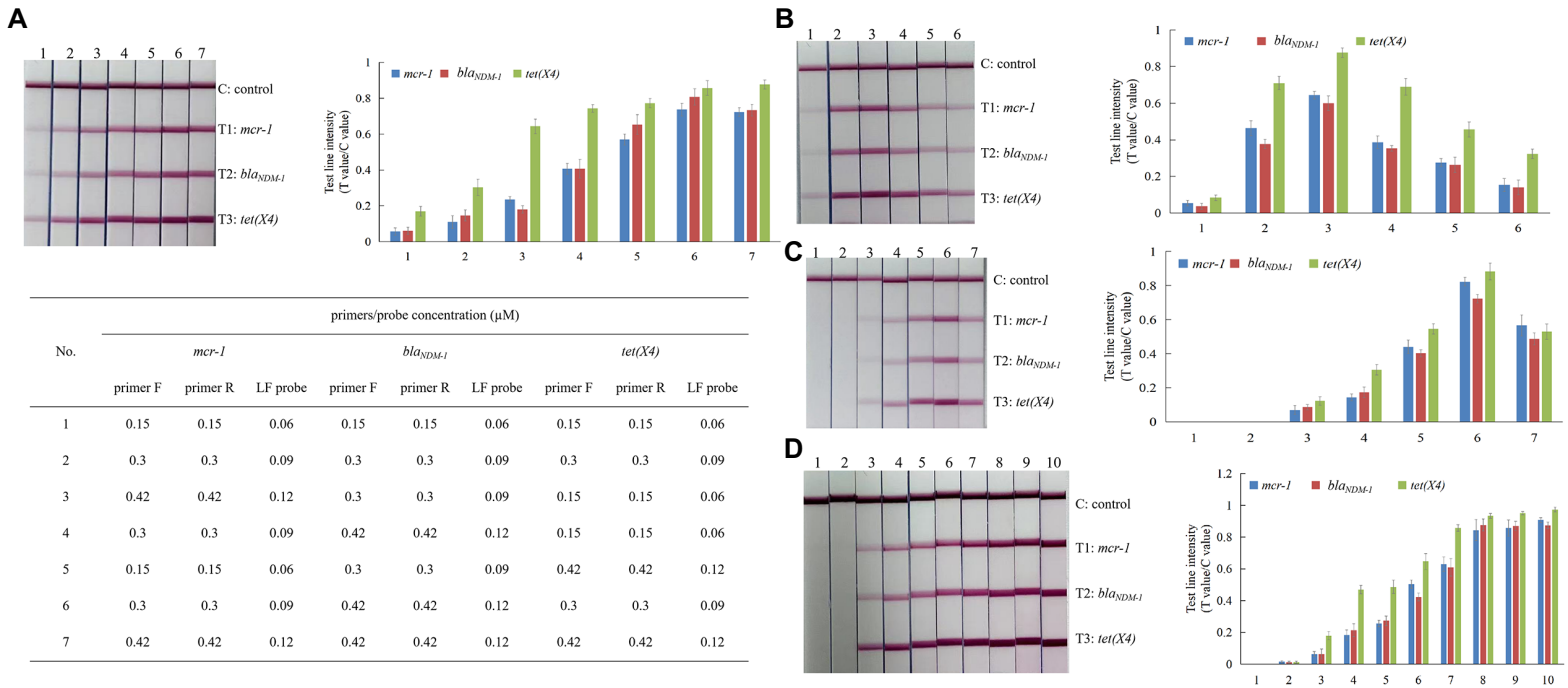
Optimization of triplex RPA amplification conditions. **(A)** Concentration of primer/LF probe; **(B)** reaction temperature (1–6: 30, 35, 37, 39, 45, 50°C); **(C)** concentration of magnesium-acetate (1–7: 0, 2.8, 5.6, 8.4, 11.2, 14, 16.8 mM); **(D)** reaction time (1–10: 0, 2.5, 5, 7.5, 10, 12.5, 15, 20, 25, 30 min).

Triplex RPA amplification conditions for simultaneous detection of *mcr-1*, *bla_NDM-1_* and *tet(X4)* using the TwistAmp nfo kit were optimized. For initial optimization, the concentration of the primer/probe set was optimized for the RPA reaction. As shown in Figure 2A, the results of RPA-LFD assays indicated that a medium concentration of primer/probe (*mcr-1* 0.30 μM/0.06 μM, *bla_NDM-1_* 0.42 μM/0.12 μM and *tet(X4)* 0.30 μM/0.09 μM) in a 50 μl reaction volume gave results similar to a high concentration of primer/probe (*mcr-1* 0.42 μM/0.12 μM, *bla_NDM-1_* 0.42 μM/0.12 μM and *tet(X4)* 0.42 μM/0.12 μM).

The RPA assay was run at incubation temperatures ranging from 30 to 50°C, and amplicons were run on LFDs to identify the optimal amplification temperature. As shown in Figure 2B, the results of RPA-LFD assays indicated that 37°C was confirmed as the optimal temperature.

Using the optimal concentrations of primer/probe sets and the optimal reaction temperature, the concentration of magnesium-acetate was tested from 0 to 16.8 mM. The results showed that the optimal concentration of magnesium-acetate was 14 mM (Figure 2C).

Finally, the RPA reaction time was validated. As shown in Figure 2D, all T1–T3 lines were visible at or after 5 min. The T/C values of T1–T3 were not significantly different at 20 min compared with 25 or 30 min. Thus, to shorten the detection time, a reaction time of 20 min was deemed sufficient.

### 3.3. Specificity of the triplex RPA-LFD assay

**Figure 3:**
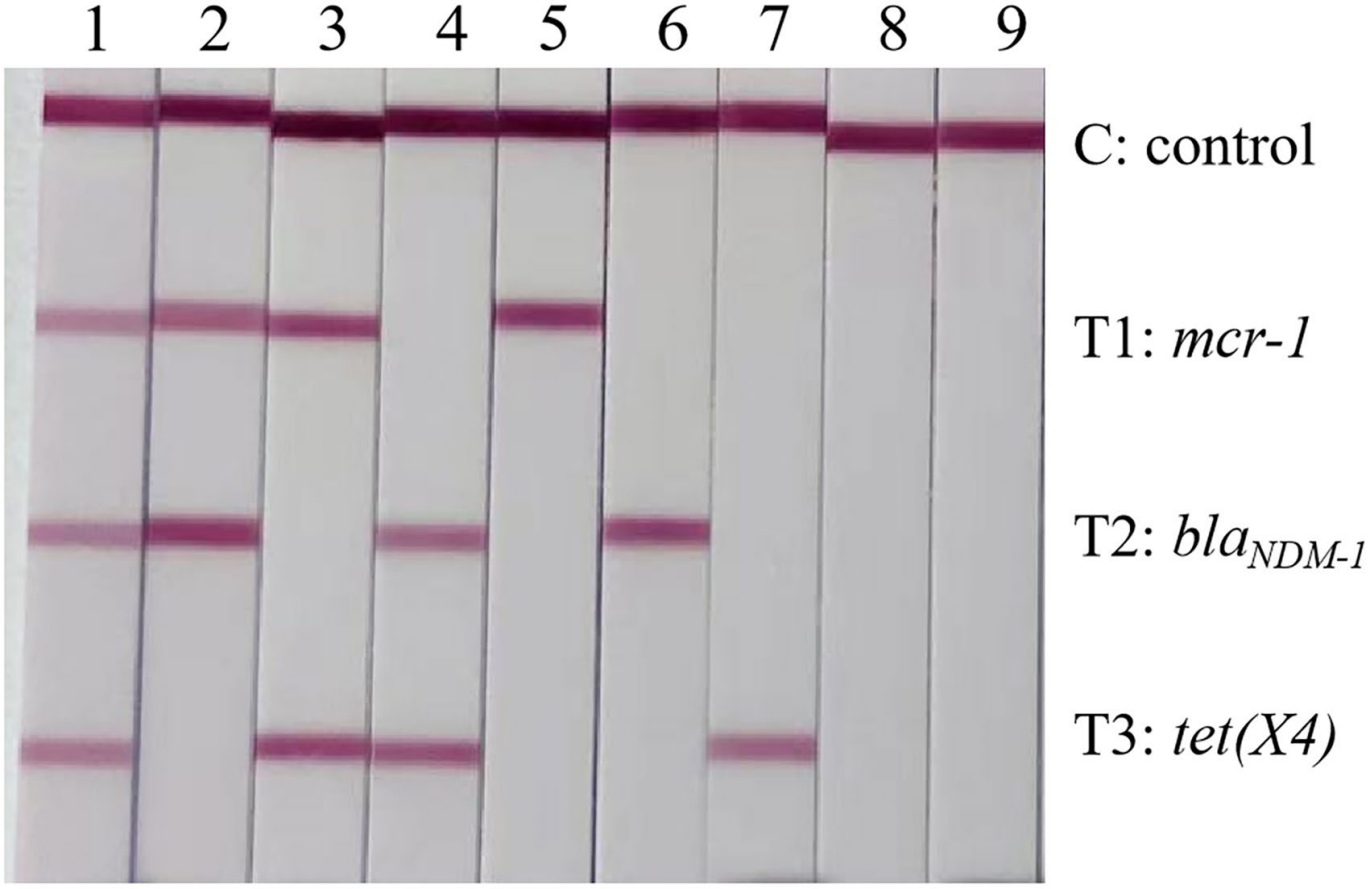
Specificity analysis of the triplex RPA-LFD assay. 1: Top10-pUC-*mcr-1 +* Top10-pUC-*bla_NDM-1_ +* Top10-pUC-*tet(X4)*; 2: Top10-pUC-*mcr-1 +* Top10-pUC-*bla_NDM-1_*; 3: Top10-pUC-*mcr-1 +* Top10-pUC-*tet(X4)*; 4: Top10-pUC-*bla_NDM-1_ +* Top10-pUC-*tet(X4)*; 5: Top10-pUC-*mcr-1*; 6: Top10-pUC-*bla_NDM-1_*; 7: Top10-pUC-*tet(X4)*; 8: ATCC25922; 9: negative control.

Standard recombinant *E. coli* strains carrying *mcr-1*, *bla_NDM-1_* or *tet(X4)* and *E. coli* ATCC25922, which does not harbor *mcr-1, bla_NDM-1_ or tet(X4)*, served for evaluating the specificity exhibited by the triplex RPA-LFD assay. Based on specificity analyses results, the triplex RPA-LFD assay performed equally well for specific samples containing the target resistance genes (Figure 3).

### 3.4. Sensitivity of the triplex RPA-LFD assay

**Figure 4:**
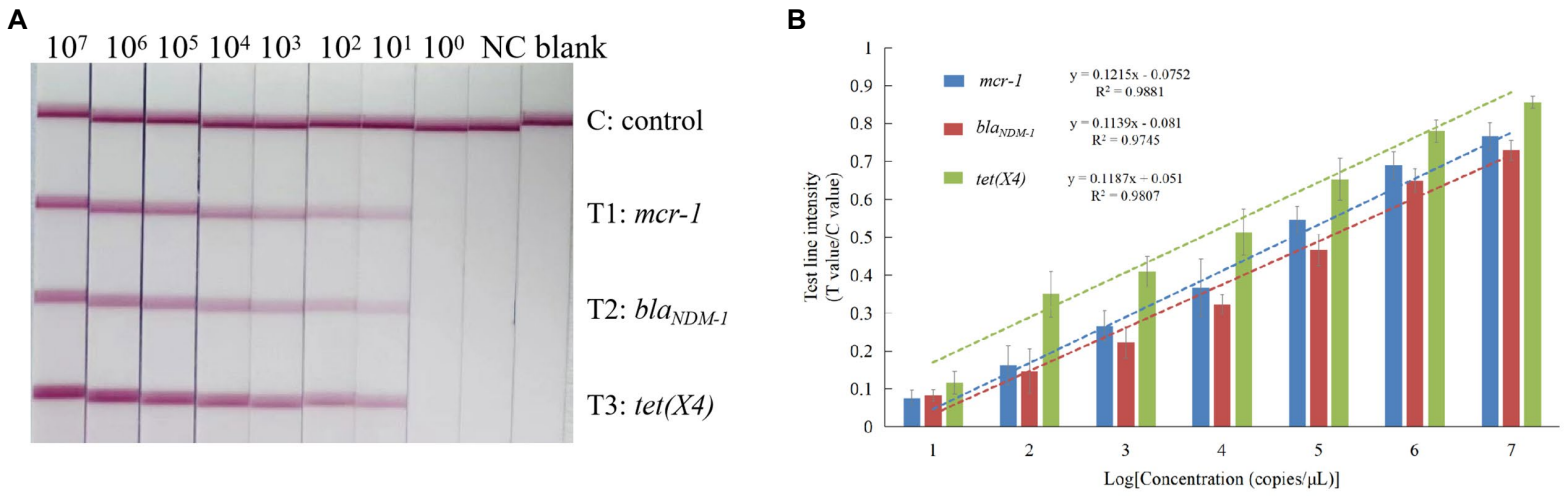
Sensitivity analysis of the triplex RPA-LFD assay. **(A)** Results of RPA-LFD assays. Blank means only the running buffer was used. **(B)** Standard curves. The standard linear equation and correlation coefficient (*R*^2^) between the T/C value and the logarithm of the copy number of target DNA (copies/μl).

The study focused on preparing the 10-fold serial dilutions of standard plasmid solutions for assessing the sensitivity exhibited by the triplex RPA-LFD assays. The concentration of the plasmids was measured, and the copy numbers of the plasmids were calculated. As shown in Figure 4A, when the concentration of the target gene was ≥10^1^ copies/μl, the T-line was visible and T values of the T1, T2, and T3 lines were measurable with the test strip reader. Hence, the detection limit of the RPA-LFD assay for all three target resistance genes was 10^1^ copies/μl.

The T/C value was positively correlated with the concentration of target DNA. Standard curves for the *mcr-1*, *bla_NDM-1_* and *tet(X4)* genes in *Enterobacteriaceae* strains were established according to the T/C values of the LFD strips and the logarithm of the copy number of the corresponding target resistance gene. As shown in Figure 4B, the results indicated that there were significant linear correlations between the T/C values and the copy number logarithm of the target DNA. The standard linear equation and correlation coefficients (*R*^2^) of the *mcr-1*, *bla_NDM-1_* and *tet(X4)* genes were *y* = 0.1215x − 0.0752 (*R*^2^ = 0.9881), *y* = 0.1139x − 0.081 (*R*^2^ = 0.9745) and *y* = 0.1187x + 0.051 (*R*^2^ = 0.9807), respectively.

### 3.5. Application analysis of the triplex RPA-LFD assay

The triplex RPA-LFD assay together with conventional PCR methods assisted in analyzing 19 different *Enterobacteriaceae* strains. As shown in Table 2, the two methods also served for detecting six *mcr-1* positive strains, three *bla_NDM-1_* positive strains, six *tet(X4)* positive strains and four *mcr-1* and *bla_NDM-1_* positive strains. The results for the target resistance genes were consistent for the two methods. The triplex RPA-LFD assay, including sample pretreatment, RPA amplification and LFD lateral flow, could be achieved within 40 min. In contrast, the PCR method, including sample pretreatment, PCR amplification and agarose gel electrophoresis, required at least 3 h. Thus, the triplex RPA-LFD assay consumes less time.

**Table 2:** Application analysis of the triplex RPA-LFD assay and PCR method.

Sample ID	Genotype	Species	Triplex RPA-LFD			PCR		
			(copies/μl)					
			*mcr-1*	*bla_NDM-1_*	*tet(X4)*	*mcr-1*	*bla_NDM-1_*	*tet(X4)*
JH-17	*tet(X4)*	*Escherichia coli*	*−*	*−*	3.5 × 10^4^	*−*	*−*	*+*
LS-62	*tet(X4)*	*Escherichia coli*	*−*	*−*	7.8 × 10^5^	*−*	*−*	*+*
TZ-118	*tet(X4)*	*Escherichia coli*	*−*	*−*	9.4 × 10^4^	*−*	*−*	*+*
HUZ-208	*tet(X4)*	*Escherichia coli*	*−*	*−*	4.6 × 10^4^	*−*	*−*	*+*
QZ-116	*tet(X4)*	*Escherichia fergusonii*	*−*	*−*	8.5 × 10^3^	*−*	*−*	*+*
NN-35	*tet(X4)*	*Klebsiella pneumoniae*	*−*	*−*	2.1 × 10^3^	*−*	*−*	*+*
JX-142	*mcr-1*	*Escherichia coli*	4.8 × 10^2^	*−*	*−*	*+*	*−*	*−*
LS-44	*mcr-1*	*Escherichia coli*	3.5 × 10^4^	*−*	*−*	*+*	*−*	*−*
LS-55	*mcr-1*	*Escherichia coli*	8.0 × 10^3^	*−*	*−*	*+*	*−*	*−*
NB-303	*mcr-1*	*Escherichia coli*	3.2 × 10^2^	*−*	*−*	*+*	*−*	*−*
HUZ-54	*mcr-1*	*Salmonella Ngor*	1.8 × 10^3^	*−*	*−*	*+*	*−*	*−*
WZ-69	*mcr-1*	*Salmonella Goldcoast*	3.8 × 10^4^	*−*	*−*	*+*	*−*	*−*
HUZ-215	*bla_NDM-1_*	*Escherichia coli*	*−*	7.8 × 10^4^	*−*	*−*	*+*	*−*
JH-51	*bla_NDM-1_*	*Escherichia coli*	*−*	5.4 × 10^3^	*−*	*−*	*+*	*−*
WZ-22	*bla_NDM-1_*	*Escherichia coli*	*−*	6.0 × 10^3^	*−*	*−*	*+*	*−*
HAZ-2	*bla_NDM-1_ + mcr-1*	*Escherichia coli*	5.2 × 10^3^	3.7 × 10^3^	*−*	*+*	*+*	*−*
HAZ-6	*bla_NDM-1_ + mcr-1*	*Escherichia coli*	1.1 × 10^2^	9.8 × 10^1^	*−*	*+*	*+*	*−*
HAZ-13	*bla_NDM-1_ + mcr-1*	*Escherichia coli*	3.5 × 10^3^	2.0 × 10^3^	*−*	*+*	*+*	*−*
HAZ-3	*bla_NDM-1_ + mcr-1*	*Klebsiella pneumoniae*	4.3 × 10^3^	7.4 × 10^3^	*−*	*+*	*+*	−

## 4. Discussion

Colistin, carbapenems and tigecycline are considered last-resort antibiotics for defense against MDR bacterial infections (Tang et al., 2021). However, these antibiotics become less effective because their respective predominant resistance genes, *mcr*, *bla_NDM_* and *tet(X)*, emerge and spread in animals, food and humans (Singh et al., 2018; Zhong et al., 2019; Anyanwu et al., 2022; Lu et al., 2022; Zhang et al., 2022). Among these last-resort antibiotics, colistin was heavily used in food-producing animal industries in China for a long period of time until it was banned as a growth promoter in 2017 (Walsh and Wu, 2016). However, colistin-resistant Gram-negative pathogens, especially *Enterobacteriaceae* pathogens, still have high presence in poultry/livestock farm environments (Liu et al., 2016; Tang et al., 2022), and the plasmid-borne resistance gene *mcr-1* is the main factor contributing to widespread colistin resistance (Arcilla et al., 2016). In addition, although the use of carbapenems, broad-spectrum β-lactam class antibiotics, is authorized only in clinics (Ahmad et al., 2019; Farhat and Khan, 2020; Nasser et al., 2020), their main resistance gene, *bla_NDM-1_*, is still frequently identified in *Enterobacteriaceae* pathogens in poultry/livestock farm environments. This is because of the extensive application of all β-lactam antibiotics (except carbapenems) in the prophylaxis and growth promotion in food-producing animal industries over the last decade in China and have provided long-term selective pressure for the *bla_NDM-1_* gene (Shen et al., 2016; Shi et al., 2021). Similarly, although the animal husbandry field has never adopted tigecycline, a third-generation tetracycline, excessively using of first- and second-generation tetracyclines may have provided a selective pressure for the *tet(X4)* gene in poultry/livestock farm environments (Tang et al., 2021). Of note, a growing number of reports indicate that *tet(X4)* is often detected together with *bla_NDM-1_* genes or *mcr-1* genes (Bai et al., 2019; Sun C. et al., 2019). Additionally, *tet(X4)*-positive strains also harboring both *mcr-1* and *bla_NDM_* genes have been found in food animal samples (Sun J. et al., 2019).

MDR *Enterobacteriaceae* pathogens in poultry/livestock farm environments are considered the most common reservoirs of the *mcr-1*, *bla_NDM-1_* and *tet(X4)* genes (Xiong et al., 2018; Xia et al., 2019; Shi et al., 2021). Besides, such ARGs may be spread along the food chain in poultry/livestock farm environments and meat production facilities, and finally be applied in community and even hospital environments (Wang et al., 2017; Shi et al., 2021). As resistance genes to last-resort antibiotics potentially threaten the food safety as well as public health, predominant ARGs such as *mcr-1*, *bla_NDM-1_* and *tet(X4)* in their common *Enterobacteriaceae* pathogen hosts should be more closely monitored along the food chain worldwide over the long-term (Shi et al., 2021). Therefore, it is in urgent need to develop a rapid, and accurate detection method to simultaneously screen and monitor multiple resistance genes to last-resort antibiotics in the field more effectively.

Multiple nucleic acid detection methods, including multiplex PCR, multiplex loop-mediated isothermal amplification (LAMP) and multiplex RPA assays, can achieve rapid, accurate and simultaneous amplification of multiple resistance genes to last-resort antibiotics in a single reaction. However, multiplex PCR assays require thermal cycling steps and specialized instruments, giving them limited application in the field (Ma et al., 2020). Similarly, multiplex LAMP assays require more target-specific primers (normally four to six) for amplification under isothermal conditions between 60 and 65°C in 40 to 60 min (Fang et al., 2018). Compared with these two methods, multiplex RPA assays require fewer target-specific primers (two primers or two primers with one probe) for amplification, lower incubation temperatures (25–43°C) and shorter incubation times (less than 30 min) and have a high tolerance to sample impurities (Lillis et al., 2014; Ma et al., 2020). Thus, in our study, a fast, innovative boil-up method for crude DNA extraction (taking around 10 min for the entire extraction process) from *Enterobacteriaceae* pathogens was used to increase the time savings of the multiplex RPA procedure. Studies have confirmed the application of multiplex RPA assays for simultaneously detecting multiple foodborne pathogens (Ma et al., 2020; Jin et al., 2022). However, studies on its detection for ARG remain less. Our study focused on explaining certain and quantitative multiplex RPA-LFD method to simultaneously identify multiple resistance genes to last-resort antibiotics in certain *Enterobacteriaceae* pathogens when the temperature remained unchanged and no specialized instruments were used, which has great potential for applications in the field.

For multiplex RPA assays for ARG detection, primer/probe concentration, incubation temperature and time and magnesium-acetate concentration are the most vital extrinsic factors contributing to the assay’s maximum efficiency (Hu et al., 2019; Ma et al., 2020). Our study first adjusted the primer/probe concentration to 0.30 μM/0.06 μM for the *mcr-1* gene, 0.42 μM/0.12 μM for the *bla_NDM-1_* gene and 0.30 μM/0.09 μM for the *tet(X4)* gene with 14 mM magnesium-acetate for obtaining an equivalent amplification for three ARGs. Second, we set the optimal time at 20 min and the temperature conditions at 37°C. This reaction time is significantly faster than that of multiplex PCR (around 4 h) and multiplex LAMP (40–60 min) assays. Furthermore, it is allowed to complete the RPA reaction in a heating block or water bath, even at ambient temperature or body temperature (Lillis et al., 2014; Wang et al., 2017).

Experiments confirm LFD as a faster and simpler tool for detecting RPA product, featuring an antibody labeled with antigen-specific gold nanoparticles, thereby ensuring that it is not needed to clean up nucleotide. The resulting visualization can be observed within 5 min (Wu et al., 2017). To achieve triplex detection in our study, we labeled each of the LF probes with biotin, Cy5 and TAMRA at the 5′ end, with a C3 spacer at the 3′ end and modified inside the LF probe with THF. We labeled all the reverse primers with digoxin at the 5′ end. We finally obtained 3 detectable double-labeled amplicons. Besides, the preparation of LFDs with three test lines served for RPA amplicon visualization (Ma et al., 2020). In general, LFD is a qualitative or semi-quantitative detection method, but the current test strip reader, which combines facile colorimetric readouts with test strips, can realize quantitative detection (Wang et al., 2022). In our study, sensitivity analyses showed a close association of color signals with template concentrations of *mcr-1*, *bla_NDM-1_* and *tet(X4)* genes in *Enterobacteriaceae* pathogens (*R*^2^ = 0.9881, *R*^2^ = 0.9745, and *R*^2^ = 0.9807, respectively). Use of the test strip reader also eliminates errors associated with assessment by eye.

Sensitivity and specificity analyses verified that RPA-LFD assays were practical for multiple resistance genes to last-resort antibiotics detection in *Enterobacteriaceae* pathogens. In our study, this assay simultaneously detected as few as 10^1^ copies/μl of *mcr-1*, *bla_NDM-1_* and *tet(X4)* genes in *Enterobacteriaceae* pathogens, with a sensitivity 10-fold higher than that of PCR (10^2^ copies/μl). Furthermore, such ARGs were specifically detected in different *Enterobacteriaceae* pathogens. These results showed that RPA-LFD is a suitable method for detecting multiple resistance genes to last-resort antibiotics in foodborne pathogens and has potential applications in the field.

## 5. Conclusion

In conclusion, the multiplex RPA-LFD method is fast, sensitive, specific and user-friendly for direct detection of the *mcr-1*, *bla_NDM-1_* as well as *tet(X4)* genes of *Enterobacteriaceae* pathogens and does not require expensive equipment. It can provide immediate monitoring results of multiple resistance genes to last-resort antibiotics in foodborne pathogens in the field in a timely manner. On this basis, we will further study the pretreatment technology of the complex sample, and establish the RPA-LFD method for direct analysis of ARGs in water, soil and feces and other environmental samples.

## Data availability statement

The original contributions presented in the study are included in the article/Supplementary material, further inquiries can be directed to the corresponding authors.

## Author contributions

CL took charge of original draft writing, data curation, and review and editing. JW was responsible for original draft writing, data curation, and software. XG, WL, DC, CZ, QY, CX, and BT together took charge of data curation and software. LP, YT, and PL acquired the funding. GH also participated in review and editing. JF and HJ participated in funding acquisition, methodology, supervision, and review and editing. All authors contributed to the article and approved the submitted version.

## Funding

This research was completed with the support of the Public Welfare Technology Application Research Project of Zhejiang Province (No. LGN22C200013), the Public Welfare Technology Application Research Project of Ningbo (No. 2022S014), the Major Science and Technology Project of Xiaoshan District (No. 2021226), the Open Fund of the Key Laboratory of Biosafety detection for Zhejiang Market Regulation (No. 2022BS002), Science and Technology Innovation Activity Plan for College Students in Zhejiang Province (No. 2022R409A057 and 2022R409A050), the National Science Foundation of China (Nos. 31901792 and 31801655), and the National Program on Key Research Project of China (No. 2019YFE0103900).

## Conflict of interest

LP was employed by Zhejiang Hongzheng Testing Co., Ltd, Ningbo, Zhejiang, China. XG was employed by Zhejiang Gongzheng Testing Center Co., Ltd, Hangzhou, Zhejiang, China. YT was employed by Hangzhou Tiannie Technology Co., Ltd, Hangzhou, Zhejiang, China.

The remaining authors declare that the research was conducted in the absence of any commercial or financial relationships that could be construed as a potential conflict of interest.

## Publisher’s note

All claims expressed in this article are solely those of the authors and do not necessarily represent those of their affiliated organizations, or those of the publisher, the editors and the reviewers. Any product that may be evaluated in this article, or claim that may be made by its manufacturer, is not guaranteed or endorsed by the publisher.

## Footnotes

^1^ https://www.ncbi.nlm.nih.gov/gene/
