# Supplementary material for: Rapid detection of multiple resistance genes to last-resort antibiotics in Enterobacteriaceae pathogens by recombinase polymerase amplification combined with lateral flow dipstick
Source: Front Microbiol. 2023 Jan 5;13:1062577. doi: 10.3389/fmicb.2022.1062577 (PMC9850091; doi:10.3389/fmicb.2022.1062577)
Supplement: Supplementary file 1 [file Table_1.docx]

**Supplementary materials:**

**Table S1 Filed isolates examined in the study**

| Isolates | Genotype | Species | Resources | Places and time | Drug-resistant phenotype^*^ |
| --- | --- | --- | --- | --- | --- |
| JH-17 | *tet(X4)* | *Escherichia coli* | Pig feces | Jinhua City, 2020 | AMP-TET-FFC-SIZ-SMZ/TMP-TIG |
| LS-62 | *tet(X4)* | *Escherichia coli* | Pig feces | Lishui City, 2020 | AMP-TET-FFC-SIZ-SMZ/TMP-TIG |
| TZ-118 | *tet(X4)* | *Escherichia coli* | Chicken feces | Taizhou City, 2020 | AMP-GEN-SPT-TET-FFC-SIZ-SMZ/TMP-  TIG |
| HUZ-208 | *tet(X4)* | *Escherichia coli* | Pig feces | Huzhou City, 2020 | AMP-SPT-TET-FFC-SIZ-SMZ/TMP-ENR-  TIG |
| QZ-116 | *tet(X4)* | *Escherichia fergusonii* | Pig feces | Quzhou City, 2021 | AMP-TET-FFC-SIZ-SMZ/TMP-  TIG |
| NN-35 | *tet(X4)* | *Klebsiella pneumoniae* | Pig feces | Nanning City, 2020 | AMP-GEN-SPT-TET-FFC-SIZ-SMZ/TMP-  TIG |
| JX-142 | *mcr-1* | *Escherichia coli* | Pig feces | Jiaxing City, 2020 | AMP-TET-FFC-SIZ-TIG |
| LS-44 | *mcr-1* | *Escherichia coli* | Chicken feces | Lishui City, 2020 | AMP-SPT-TET-FFC-SIZ-SMZ/TMP-ENR-  OFX-TIG |
| LS-55 | *mcr-1* | *Escherichia coli* | Chicken feces | Lishui City, 2020 | AMP-SPT-TET-FFC-SIZ-SMZ/TMP-ENR-  OFX-TIG |
| NB-303 | *mcr-1* | *Escherichia coli* | Chicken feces | Ningbo City, 2020 | AMP-GEN-SPT-TET-FFC-SIZ-SMZ/TMP-  ENR-TIG |
| HUZ-54 | *mcr-1* | *Salmonella Ngor* | Duck feces | Huzhou City, 2020 | AMP-TET-FFC-SIZ-SMZ/TMP-ENR-  OFX-TIG |
| WZ-69 | *mcr-1* | *Salmonella Goldcoast* | Duck feces | Wenzhou City, 2020 | AMP-GEN-TET-FFC-SIZ-TIG |
| HUZ-215 | *bla_NDM-1_* | *Escherichia coli* | Pig feces | Huzhou City, 2020 | AMP-GEN-SPT-TET-FFC-SIZ-SMZ/TMP-  CEF-ENR-OFX-COL |
| JH-51 | *bla_NDM-1_* | *Escherichia coli* | Pig feces | Jinhua City, 2020 | AMP-GEN-SPT-TET-FFC-SIZ-SMZ/TMP-  ENR-OFX-COL |
| WZ-22 | *bla_NDM-1_* | *Escherichia coli* | Pig feces | Wenzhou City, 2020 | AMP-GEN-SPT-TET-FFC-SIZ-SMZ/TMP-  CEF-ENR-OFX-COL |
| HAZ-2 | *bla_NDM-1_+mcr-1* | *Escherichia coli* | Pork meat | Hangzhou City, 2021 | AMP-TET-FFC-SIZ-TIG-GEN-SPT-  SMZ/TMP-CEF-ENR-OFX-COL |
| HAZ-6 | *bla_NDM-1_+mcr-1* | *Escherichia coli* | Pork meat | Hangzhou, City 2021 | AMP-TET-SPT-FFC-SIZ-SMZ/TMP-TIG-CEF-ENR-OFX-COL |
| HAZ-13 | *bla_NDM-1_+mcr-1* | *Escherichia coli* | Pork meat | Hangzhou City, 2021 | AMP-SPT-TET-FFC-SIZ-SMZ/TMP-ENR-  TIG-GEN-CEF-OFX-COL |
| HAZ-3 | *bla_NDM-1_+mcr-1* | *Klebsiella pneumoniae* | Pork meat | Hangzhou, 2021 | AMP-GEN-SPT-TET-FFC-SIZ-  SMZ/TMP-CEF-ENR-OFX-COL |
| ATCC25922 * | */* | *Escherichia coli* | / | CICC, 2019 | / |
| Top10-pUC-*mcr-1* | *mcr-1* | *Escherichia coli* | / | Construction in our lab | / |
| Top10-pUC-*bla_NDM-1_* | *bla_NDM-1_* | *Escherichia coli* | / | Construction in our lab | / |
| Top10-pUC-*tet(X4)* | *tet(X4)* | *Escherichia coli* | / | Construction in our lab | / |

*Drug-resistant phenotype were determined by antimicrobial susceptibility test and bacteria obtained resistance against ≥ 3 categories of antibiotics were regarded as MDR. AMP: Ampicillin; TET: Tetracycline; FFC: Florfenicol; SIZ: Sulfisoxazole; SMZ/TMP: Sulfamethoxazole/Trimethoprim; TIG: Tigecycline; GEN: Gentamicin; SPT: Spectinomycin; ENR: Enrofloxacin; OFX: Ofloxacin; CEF: Ceftiofur; COL: Colistin; CAZ: Ceftazidime; MEM: Meropenem; AMC: Amoxicillin/clavulanic acid; ATCC: American Type Culture Collection, Virginia, USA; CICC: China Center of Industrial Culture Collection, Shanghai, China. *: *Escherichia coli* ATCC25922 was always used as quality control reference strains in all of the antimicrobial susceptibility tests.
